# Parliament and the Professions

**Published:** 1919-11-29

**Authors:** 


### PARLIAMENT AND THE PROFESSIONS.

### Employment of Demobilised Medical Men.

GENERAL CROFT having raised the question of the employment of medical men who on joining the Army sacrificed their practices, was assured by Mr. Churchill that every sympathy was felt for such cases, and that, when part-time services of civil medical practitioners are required, the Military Commands have been asked to use every endeavour to employ demobilised officers who have served overseas. It is understood also that the Ministry of Health endeavour to bring about preference to such candidates for appointments under local authorities.

### Indian Medical Services Officers' Leave.

MR. MONTAGUE informed Mr. Perkins that the leave of a number of I.M.S. officers in Mesopotamia is overdue, and no effort is spared to remedy this condition, which is not peculiar to Mesopotamia. I.M.S. officers in civil employment in Mesopotamia, it is hoped, will be replaced from this country before very long.

### Infectious Diseases in London.

DR. ADDISON stated that the number of cases notified in the county of London recently were as follows:

| | September. | October. |
|---|---|---|
| Scarlet fever ... ... ... | 1,205 | 2,017 |
| Diphtheria ... ... .. | 775 | 1,074 |

Admissions to Metropolitan Asylums Board hospitals were:

| | September. | October. |
|---|---|---|
| Scarlet fever ... ... ... | 1,100 | 1,502 |
| Diphtheria ... ... ... | 750 | 925 |

Some difficulty as to accommodation during the last six weeks has arisen because of the substantial increase in cases, the fact that the military had not evacuated all the hospitals, and the difficulty in securing all the nurses and domestic staff required, which is accentuated by the reduction of working hours. On November 13 there were 1,589 more cases in M.A.B. hospitals than on any date last year.

### Maintenance of Hospitals.

COLONEL NEWMAN asked the Ministry of Health whether the Government have any intention of converting the hospitals hitherto maintained by voluntary contributions into institutions partially or wholly assisted from public funds. Dr. Addison said that the step suggested would not be within the power of the Ministry of Health, without legislation, in respect of any hospitals not willing to receive such subsidies.

### R A M.C. Alleged Discontent.

SIR WATSON CHEYNE having, in a question to the Secretary for War, raised the point of the alleged dissatisfaction of temporary R.A.M.C. officers in India on account of the manner in which their contracts of service are being interpreted, was informed by Mr. Churchill that he did not know of any discontent. Temporary officers serving in India were offered a new contract at 800 rupees per mensem if they would undertake to serve in that country for another year. Those eligible for demobilisation, and who did not sign the new contract, but whose services could not immediately be spared, were granted 750 rupees per month, with effect from July 1, 1919.

### Physical Education of Children.

MR. FISHER stated that special grants are paid by the Board of Education in aid of expenditure of local education authorities on the employment of competent persons to organise and supervise the physical training of children in public elementary schools. This applies not only to counties but to the areas of all authorities for elementary education, and fifty-one such authorities are now employing recognised organising superintendents.

### Treatment by Psycho-Therapy.

MR. MACQUISTEN asked the Pensions Minister whether he proposes to establish throughout the country clinics for the treatment by means of psycho-therapy of functional nervous affections (including so-called shell-shock), and, if so, he will see that pensioners recommended are informed of the nature of the treatment, in which hypnotic suggestion is used, that they will not be prejudiced should they decline to undergo it, and that pensioners will have offered them the alternative of ordinary therapeutic treatment. Sir L. Worhington-Evans stated that such clinics are to be formed, and reminded the questioner that psycho-therapy is a comprehensive term, including many forms of treatment, of which hypnotic suggestion is the form least frequently used. If in any case hypnotism should be proposed as the most desirable form of treatment the man would be perfectly free to refuse it, and no penalty whatever would attach to his refusal.

